# Enhanced Thermal Conductivity and Dielectric Performance of CMZBS–Glass–Ceramic Composites with AlN Whisker Incorporation for LTCC Applications

**DOI:** 10.3390/ma18040857

**Published:** 2025-02-15

**Authors:** Tiange Xue, Xinqing Su, Shixiang Yu, Meng Meng, Xinya Xu, Jinqi Xin, Jinjin Ran

**Affiliations:** College of Materials Science and Technology, Nanjing University of Aeronautics and Astronautics, Nanjing 211106, China; yjyydo@nuaa.edu.cn (T.X.); shixiangyu_nuaa@163.com (S.Y.); mengmeng7@nuaa.edu.cn (M.M.); xxy_0320@nuaa.edu.cn (X.X.); xinjinqi@nuaa.edu.cn (J.X.); rjj2024@nuaa.edu.cn (J.R.)

**Keywords:** LTCC, AlN whiskers, dielectric properties, thermal properties

## Abstract

In this work, a small amount of AlN whiskers (ranging from 2 wt.% to 8 wt.%) was incorporated into CaO-MgO-ZnO-B_2_O_3_-SiO_2_ (CMZBS)–glass/Al_2_O_3_ composites so as to obtain glass-ceramics with a thermally conductive network through sintering between 700 °C and 1000 °C. Special attention was given to the densification behavior, dielectric properties, and thermal conductivity of CMZBS/Al_2_O_3_/AlN–glass–ceramic composites with varying AlN whisker contents. According to the results, composites with desirable thermal, mechanical, and dielectrical properties were successfully fabricated. Notably, the composites containing 6 wt.% AlN whiskers, sintered at 800 °C, exhibited the most optimal comprehensive properties (dielectric constant of 7.06, dielectric loss of 383 × 10^−5^, thermal expansion coefficient of 6.40·10^−6^/K, flexural strength of 180 MPa, and thermal conductivity of 5.17 W/(m·K)). Given these attributes, this CMZBS/Al_2_O_3_/AlN composite holds great potential for applications in LTCC (low-temperature co-fired ceramic).

## 1. Introduction

Since their discovery in 1982, low-temperature co-fired ceramics (LTCCs) have been known for their excellent thermal conductivity and dielectric properties [1,2,3]. Over the following decades, LTCC technology has found widespread application in various areas such as wireless communication, automotive engineering, and the aerospace industry [4,5,6,7]. LTCCs play an important role in fabricating substrates for high-density integrated circuit packaging and the miniaturization of electronic devices. In this respect, the accumulation of heat has become a serious issue that should be urgently addressed. Traditional LTCC–glass–ceramic composites, which often contain a significant amount of glass with low thermal conductivity, face stringent thermal conductivity requirements to be suitable for high-performance electronic packaging applications [8,9].

Integrating highly thermally conductive ceramic particles into the glass matrix is a viable strategy to improve the thermal conductivity of LTCC composites [10]. Notably, AlN ceramic stands out as a potential material for LTCC applications due to its superior thermal conductivity (319 W m^−1^ K^−1^), a low thermal expansion coefficient (4.5·10^−6^/K) that aligns well with silicon, favorable dielectric properties (ε_r_ = 8.8, tanδ = 5–10 × 10^−4^) [11,12,13], and other outstanding mechanical, chemical, electrical, and high-temperature physical properties [14,15]. Therefore, significant research has been dedicated to the incorporation of AlN as a thermally conductive filler [16]. Nevertheless, the improvement in composite thermal conductivity is limited because the ceramic particles, which are added to enhance heat transfer, are insulated by the low-conductivity glass phase. Among the strategies to improve the thermal characteristics of LTCCs, the incorporation of low-dimensional materials with high aspect ratios as thermally conductive fillers (e.g., whiskers [16,17], fibers [18], nanotubes [18,19], and nanosheets [20,21]) is being extensively researched with the aim of constructing a three-dimensional network structure [22]. For instance, Ma et al. [16] achieved an increase in thermal conductivity of CMBS–glass/AlN–ceramic composites by 57.98% through the addition of 14 vol.% β-Si_3_N_4_ whiskers, which resulted in the permittivity of 6.5 and the dielectric loss of 0.16% at 1 MHz, alongside good flexural strength (226 MPa). Feng et al. [23] added 20 wt.% BN to B_2_O_3_–Bi_2_O_3_–SiO_2_–ZnO (BBSZ) glass/Al_2_O_3_ tape-cast green sheets, forming a 3D heat conduction pathway, which effectively enhanced thermal conductivity while reducing permittivity to ε_r_ = 3.9672 and dielectric loss to tanδ = 1.49 × 10^−3^ at 20 GHz. Wang et al. [18] investigated the impact of various one-dimensional high-thermal-conductivity materials on enhancing the thermal conductivity of Al_2_O_3_/ZBNS–glass composites. The results showed that incorporating copper fibers into the Al_2_O_3_/glass composite remarkably enhanced its thermal conductivity, achieving a nearly 500% improvement, with values up to 38.9 W/(m·K). Yang et al. [21] incorporated 4 wt.% of hexagonal boron nitride (h-BN) into the AlN/CaCO_3_-MgO-B_2_O_3_-SiO_2_-Li_2_CO_3_–glass composites and exposed them to hot pressing. This enabled them to increase their thermal conductivity from 4.75 to 10.3 W/(m·K), as well as to achieve the optimal permittivity and dielectric loss (ε_r_ = 5.96 and tanδ = 4.77 × 10^−4^, respectively).

Because of its outstanding mechanical, chemical, electrical, thermal, and high-temperature physical properties [14,15], aluminum nitride (AlN) holds great potential in the field of LTCC materials [24,25,26]. The introduction of nitride whiskers is anticipated to markedly improve both the thermal and dielectric properties of oxide-based LTCCs [24,25,26]. In view of the above, CMZBS/Al_2_O_3_/AlN-whisker–glass–ceramic composites with high densities and excellent overall performance were prepared in this study. The introduction of a small amount of AlN whiskers (2 wt.% to 8 wt.%) was shown to be a reliable way to optimize the thermal and dielectric characteristics of ceramics while retaining the intrinsic parameters of the composites.

## 2. Experimental

### 2.1. Preparation of Composites

CaO-MgO-ZnO-B_2_O_3_-SiO_2_ (CMZBS) glass (the corresponding composition is given in Table 1) was used in the present study.

The glass was prepared according to the procedure below. First, raw materials were weighed conforming to the stoichiometry ratio and ball-milled for 6 h. After drying in the quartz crucible, the material was exposed to high-temperature melting at 1450 °C for 2 h. The liquid molten glass was then poured into a large amount of deionized water and quenched to obtain the glass slag. After drying, the glass slag was crushed with a jaw crusher until a coarse powder state, finally ball-milled for 24 h and dried for use.

The characteristics of the ceramic fillers utilized in this study are presented in Table 2.

The prepared CMZBS glass, Al_2_O_3_ powder, and AlN whiskers were weighed according to the formulae in Table 3 and then mixed in anhydrous ethanol by using the rolling mill for 6 h. After drying, PVA solution was added to promote granulation, and the resulting mixture was sieved to obtain spherical powders within the 60–80 mesh range.

After the granulation of GO50 and GOW series composites, the corresponding powders were pressed and de-molded to obtain blanks whose specifications varied according to the tests to be carried out. The samples were labeled as GO50 and GOWx (x = 2, 4, 6, and 8), while x means the mass fraction of AlN whiskers added; meanwhile, GO50 is the sample without AlN whiskers. In particular, samples with diameters of 8 mm and thicknesses of 3 mm were used for shrinkage experiments. Those with diameters of 15 mm and thicknesses of 7.5 mm were employed in the measurements of dielectric constant and dielectric loss. Specimens with diameters of 15 mm and thicknesses of 2 mm served for the XRD and thermal conductivity analyses. Finally, samples with dimensions of 50 mm (length) × 3 mm (width) × 4 mm (thickness) were separated for flexural strength, fracture morphology, and thermal expansion studies.

Different samples of green bodies are first heated at a rate of 2 °C/min to 600 °C and held at this temperature for two hours to decompose and burn the PVA introduced during the granulation process. Subsequently, the samples are heated to the sintering temperature at a rate of 5 °C/min, and after holding at this temperature for another two hours, they are allowed to cool down in the furnace to complete the sintering process.

### 2.2. Characterization

LTCC–glass–ceramic composites, when utilized as substrates, must possess superior thermal conductivity, mechanical strength, and dielectric characteristics. In this work, flexural strength characteristics of specimens were assessed via the three-point bending tests using an electric tensile-compressive strength testing machine (Zhiqu Precision Instrument Co., Model ZQ-990LB, Huai’an, China). The Archimedes’ principle was employed to determine the bulk densities of ceramics whereby the samples were immersed in the boiling deionized water for 2–4 h to completely expel air from the macro-pores. After the removal from the water, the volume densities of specimens were evaluated. Differential thermal analysis (DTA, PerkinElmer Pyris DTA 7, Shelton, CT, USA) technique by ramping up the temperature from room temperature to 1000 °C at a heating rate of 10 °C/min was used to analyze the characteristic temperatures and exothermic/endothermic phenomena during the heating process of the samples under an air atmosphere. The sintering curves and coefficients of thermal expansion (CTE) of the samples were measured using a dilatometer (DIL, NETZSCH DIL 402C, Selb, Germany) by heating the samples from room temperature up to 1000 °C at a heating rate of 5 °C/min. The test curves were corrected by using the calibration files for the alumina standards. The X-ray diffraction (XRD, Bruker D8 Advance, Billerica, MA, USA) analysis using Cu Kα radiation was performed on glass powders and sintered ceramic composites within the 2θ scan range of 20° to 80°. The microstructures of the sintered sheets were observed via a scanning electron microscope (SEM, TESCAN LYRA3 GM, Brno, Czech Republic). The thermal conductivity of the samples was determined using a laser-flash analyzer (NETZSCH LFA467, Germany). The dielectric properties of the samples were tested using a vector network analyzer (VNA, Agilent N5230A, Santa Clara, CA, USA).

## 3. Result and Discussion

### 3.1. CMZBS Glass

A well-chosen glass phase facilitates the densification of glass/ceramic composites during the sintering process, which in turn improves their ultimate properties. The CaO-B_2_O_3_-SiO_2_ (CBS) glass system exhibits excellent compatibility with alumina ceramics, enabling the precise tuning of the final glass’s properties through compositional adjustments [4,27,28,29]. Besides that, CaO and MgO are recognized as effective sintering aids for AlN [16,30], while the addition of a moderate amount of ZnO improves the chemical stability of the glass [31,32]. In this study, the CMZBS glass (containing 25 wt.% CaO, 10 wt.% MgO, 10 wt.% ZnO, 20 wt.% B_2_O_3_, and 35 wt.% SiO_2_) was produced by introducing specific concentrations of MgO and ZnO into conventional CBS glass.

**Figure 1:**
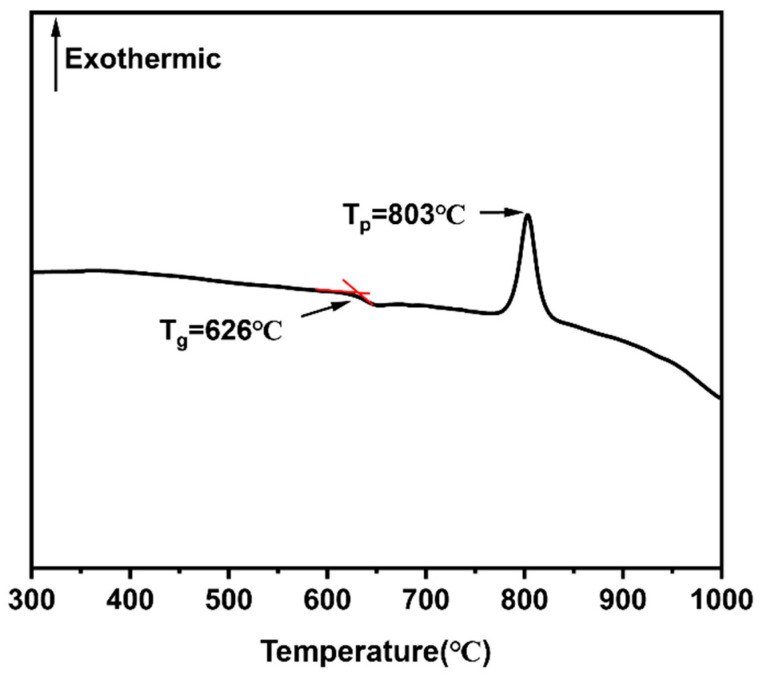
DTA curve of CMZBS glass.

Figure 1 depicts the DTA curve of the CMZBS glass employed in this study, revealing a glass transition temperature (T_g_) at 626 °C. Such a low T_g_ value is advantageous for sintering as it significantly reduces thermal stresses, ensuring the structural integrity of the final product. Additionally, it promotes efficient densification, which in turn reduces energy consumption during the sintering process. These characteristics render the CMZBS glass an excellent candidate for fabricating durable glass–ceramic composites that exhibit superior mechanical properties. Moreover, a crystallization peak observed at 803 °C signifies the beginning of crystallization within the glass matrix. The nucleation and growth of microcrystals contribute to the material ordering, which reduces phonon scattering and increases the mean free path of phonons [33]. This, in turn, increases the thermal conductivity of the composite. Simultaneously, the enhanced crystallinity is expected to decrease the dielectric properties [34,35,36], positioning the CMZBS/Al_2_O_3_/AlN-whisker composite as a promising candidate material for applications demanding high thermal and electrical performance [37].

### 3.2. Sintering Behavior of Composite Materials

**Figure 2:**
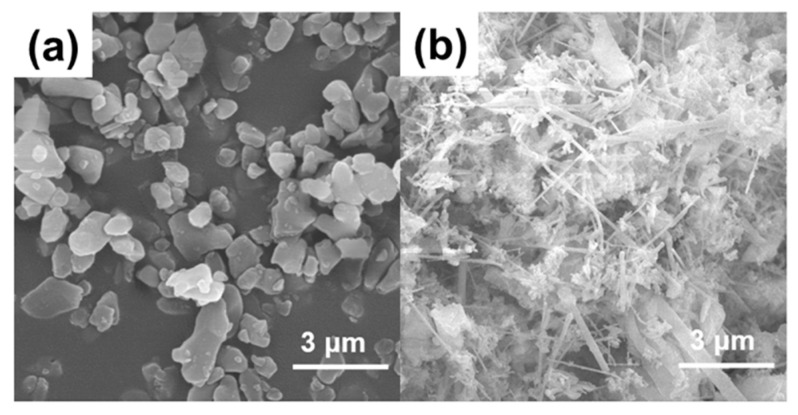
Microscopic morphology of (**a**) Al_2_O_3_ powder and (**b**) AlN whiskers.

Figure 2 displays the microscopic morphologies of Al_2_O_3_ powder and AlN whiskers. The Al_2_O_3_ particles exhibit a predominately polygonal shape and maintain a uniform size. The AlN whiskers, characterized by their long, fibrous structure, readily interweave to form a three-dimensional network within the composite material, which is expected to facilitate heat conduction.

**Figure 3:**
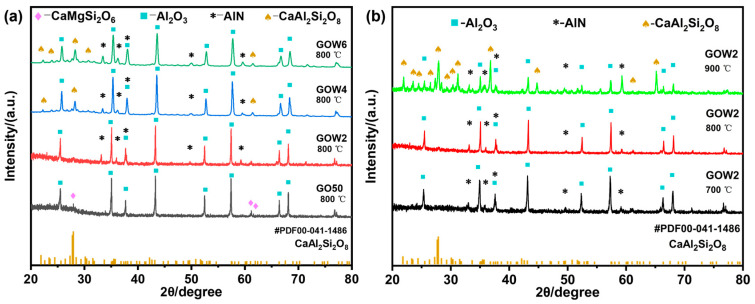
XRD images of (**a**) different composites at 800 °C and (**b**) GOW2 after sintering at different temperatures.

Figure 3a displays the XRD profiles for the GO50 and several GOW composites after a 2 h annealing process at 800 °C. Initially, the diopside (CaMgSi_2_O_6_; PDF #97-001-7043) crystalline phase is present in the GO50 sample. However, the introduction of AlN whiskers inhibits the formation of the diopside phase, as observed in the GOW2 sample. As the AlN whisker content further increases, the XRD patterns of the GOW4 sample begin to show the characteristic peaks of anorthite (CaAl_2_Si_2_O_8_; PDF #00-041-1486), and these peaks become more pronounced in the GOW6 sample. This observation suggests that AlN whiskers may act as nucleation sites for anorthite, thereby facilitating the crystallization of this phase within the composite material. Crystallization is a common phenomenon in LTCC composites, which is consistent with what has been reported in the review literature. Precrystallization is a common phenomenon in LTCC composites, which is consistent with what has been reported in the review literature [38]. The precipitated crystalline phases will have a significant impact on the thermal properties [33] in addition to contributing positively to the dielectric properties [34,35,36].

Figure 3b shows the XRD patterns of the GOW2 sample after annealing at different temperatures for 2 h. It can be seen that no new crystalline phases form when the sample is annealed at 700 °C and 800 °C, and there is no significant difference between the XRD patterns of the two samples. However, when the annealing temperature is increased to 900 °C, the GOW2 sample develops anorthite crystalline phase. As shown in Figure 1, the CMZBS glass used in this work exhibits a clear crystallization peak only around 803 °C and does not undergo secondary crystallization. However, in the composite materials, the crystallization behavior of the glass phase changes significantly, and a clear secondary crystallization occurs after 900 °C.

**Figure 4:**
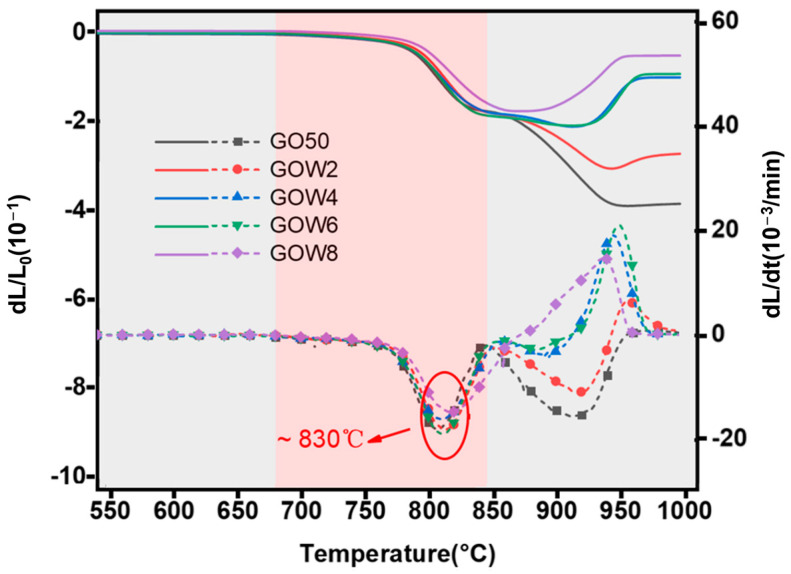
Thermal expansion (solid lines) and contraction rate (dashed lines) of GO50 and GOW series materials.

The thermal expansion curves of sintered GO50 and GOWx samples are displayed in Figure 4. The solid lines represent the extent of change, and the dashed lines indicate the rate of change in the one-dimensional direction for the GO50 and GOWx composite materials during the sintering process. According to the plots, the shrinkage began at 680 °C, reaching a maximum at 830 °C (highlighted in red circles), whereby the absolute values of peak rates for different whisker contents ranged from 15 to 20 × 10^−3^/min. As shown in Figure 3, AlN whiskers may promote the crystallization of the composite materials, leading to secondary crystallization after 900 °C. Furthermore, the higher the AlN whisker content, the more significant the promotion of both primary and secondary crystallization. Crystallization typically hinders densification, which ultimately results in differences in densification behavior among the various samples. Below 850 °C, the sintering behaviors of various samples were relatively similar, all of these composites showed some degree of shrinkage, as shown in red in Figure 4. However, when the temperature exceeds 850 °C, there is a clear difference in the densification behavior of the GOWx and GO50 samples. The GO50 samples continued to shrink, whereas the GOW2 sample began to shrink after 850 °C but expanded slightly around 950 °C due to secondary precipitation caused by the addition of AlN whiskers and then stabilized. When the AlN whisker content is increased to 4 wt.% or more, the samples swell significantly after 850 °C. Specifically, the swelling behavior of GOW4 and GOW6 is similar, and the rate of swelling peaks around 930 °C, while GOW8 swells over a wider range of temperatures and has a greater final swelling rate. This also reflects the promotion of AlN on the precipitation behavior.

**Figure 5:**
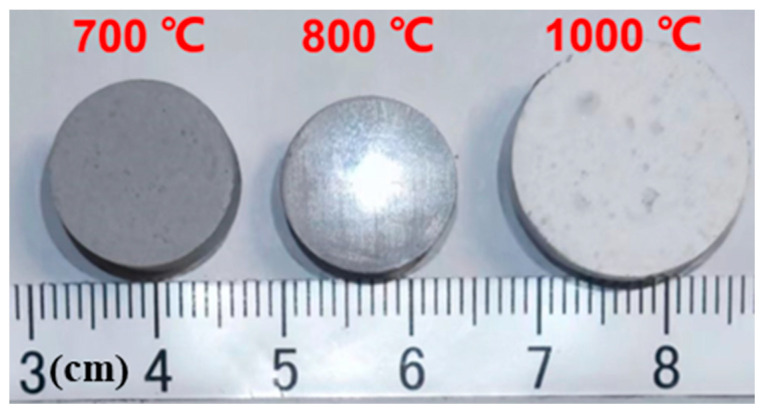
Digital image of GOW4 samples sintering at different holding temperatures.

Figure 5 displays the final digital photos of GOW4 disk samples (original diameter is 15 mm) after 2 h of sintering at different holding temperatures (700 °C, 800 °C, and 1000 °C). As seen from the images, the samples held at 700 °C were under-sintered, those held at 800 °C exhibited shrinkage, and those held at 1000 °C experienced significant expansion. These roughly reflect the trend of volume change in GOW4 during sintering, and from the results we can see that sintering at a higher temperature like 1000 °C may lead to a decrease in the density of the samples.

**Figure 6:**
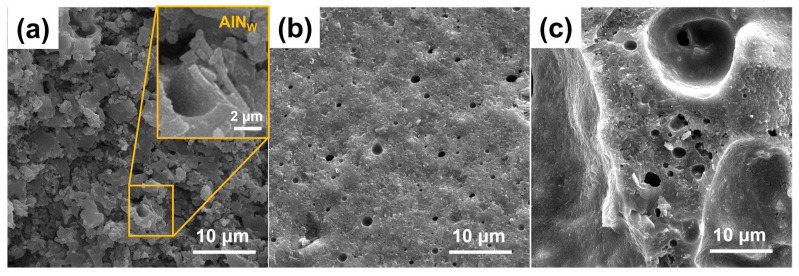
SEM images of GOW4 sintered at (**a**) 700 °C, (**b**) 800 °C, and (**c**) 1000 °C.

Figure 6 displays the SEM images of the GOW4 sample after sintering for 2 h at various temperatures, highlighting the factors that result in the observed macroscopic volume changes. As shown in Figure 6a, the insufficient formation of the liquid phase during the glass sintering at 700 °C resulted in the granular texture observed in the GOW4 sample. The overall density of the material remained inadequate.

As soon as the temperature rose to 800 °C (Figure 6b), the glass generated an adequate amount of liquid phase, allowing ceramic particles to be thoroughly wetted and rearranged. The process of wetting and particle rearrangement plays a pivotal role in enhancing the density of the final product, promoting the encapsulation of AlN whiskers within the glass matrix, and facilitating the exclusion of pores. Consequently, the increase in density leads to a noticeable reduction in macroscopic volume.

**Figure 7:**
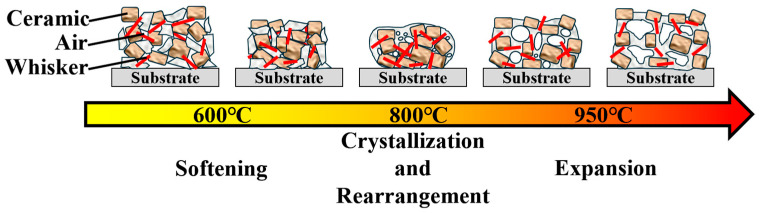
Schematic diagram of the sintering process of GOW series materials.

When the temperature further increased to 1000 °C (Figure 6c), the GOW4 sample experienced abnormal expansion, internally developing a porous structure, which caused a dramatic increase in macroscopic volume. This phenomenon is associated with crystallization. The density of anorthite is 2.76 g/cm^3^ [39], which is slightly lower than the density of the composite, and the precipitation of a large number of crystalline phases also leads to the expansion of the composite. In addition, the originally highly fluid glass phase precipitated a large amount of crystalline phases, leading to a significant decrease in fluidity [40]. The premature closure of the pores prevents the escape of air, and the two causes eventually combine to cause the whole volume to expand. As can be seen in Figure 4, the densification of the samples used in this work started to be affected around 850 °C, and by about 930 °C, the densification process of all samples had ceased. This process is schematically illustrated in Figure 7.

Thus, the density of the above composites was greatly influenced by the holding temperature, whereby too high or too low sintering temperatures might have resulted in the deviation from the requirements for LTCC applications.

### 3.3. Mechanical and Thermal Properties of Composite Materials

**Figure 8:**
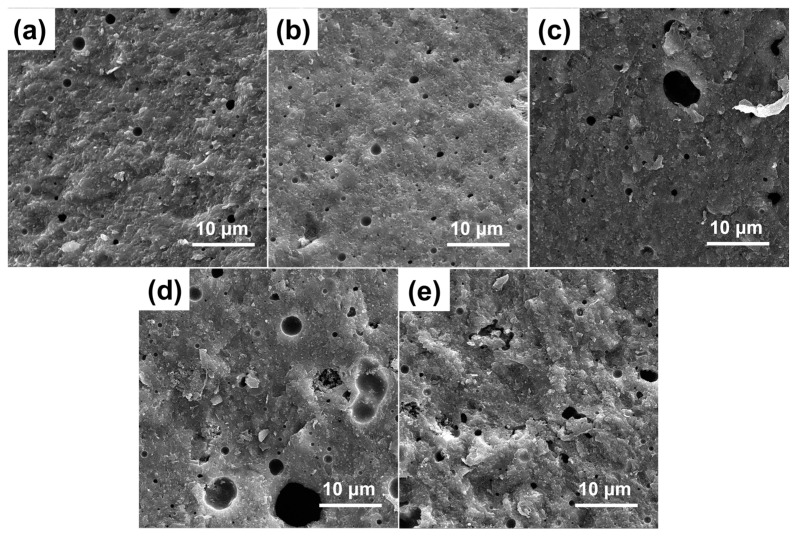
SEM images of (**a**) GO50, (**b**) GOW2, (**c**) GOW4, (**d**) GOW6, and (**e**) GOW8.

Figure 8 illustrates the fracture surface micromorphology of specimens with varying AlN whisker concentrations after being held at 800 °C for a duration of 2 h. Since the AlN whiskers promote the crystallization of CMZBS glass at high temperatures, when the whisker content increases, the number and diameter of pores in the composites increase, and thus the fracture surface will gradually become loose and rough. The increase in pore quantity and diameter might have led to a decrease in the overall density of the composites, which in turn affected their mechanical properties. Additionally, these pores could be accelerating the material’s fracture process. Thus, the concentration of added AlN whiskers must be carefully optimized to balance the performance enhancement they provide with the potential drawbacks of increased porosity and structural looseness.

**Figure 9:**
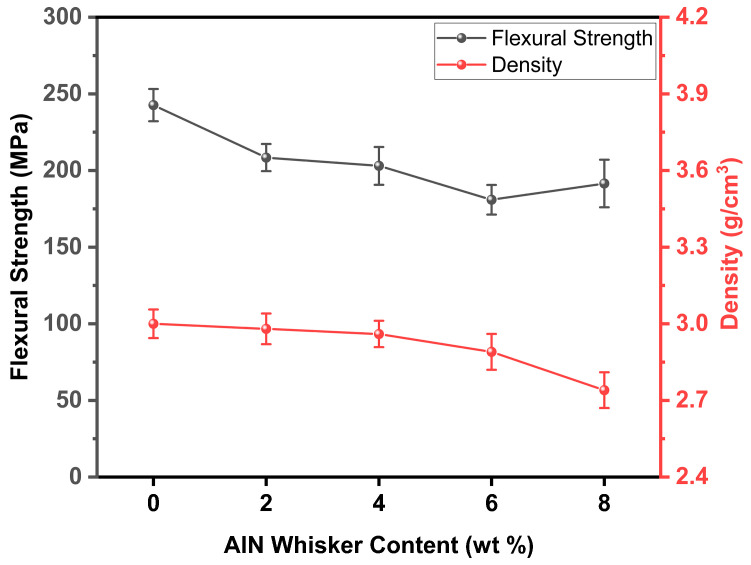
The mechanical properties of composite materials.

Figure 9 presents the mechanical properties of the composite materials. The strength of the composites gradually decreases with increasing AlN whisker content and only slightly recovers at GOW8. And it is evident that the addition of AlN whiskers led to a reduction in the density of the composites. The main reason for this is that AlN whiskers promote the crystalline precipitation behavior of the composites; the precipitated crystalline phases greatly increase the viscosity of the glass in the molten state, hindering the densification of the material, and the density of these crystalline phases is lower than that of the original phases of the composites. The crystallization became more pronounced when the whisker concentration increased. For instance, the GOW8 specimen exhibited a more pronounced and significant decrease in density when compared with the other GOW series composites. Moreover, since the pores may cause stress concentration, the increasing porosity is likely to exert a negative impact on the fracture strength of the composite materials.

**Figure 10:**
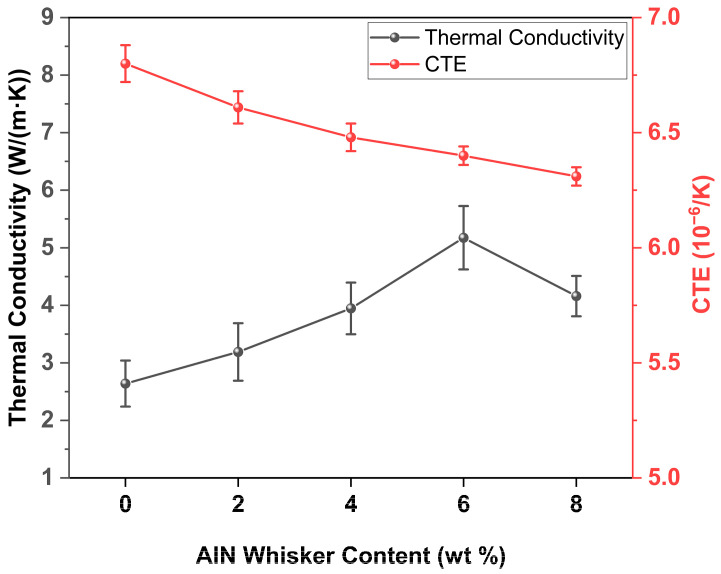
Thermal conductivity and the CTE (200 °C) of composite materials.

Figure 10 presents the thermal conductivity and coefficient of thermal expansion (CTE, at 200 °C) of the composite materials. The data indicates that the CTE of the composites decreased monotonically with the addition of AlN whiskers. This reduction is primarily due to the formation of pores, which disrupt the continuous path for heat transfer. However, the thermal conductivity of the composites showed a non-monotonic trend with increasing AlN whisker content. In the case of the GO50 sample, the thermal conductivity was 2.64 W/(m·K). This was because the Al_2_O_3_ particles were isolated by a glass phase with relatively low thermal conductivity, hindering efficient heat transfer. Upon increasing the AlN whisker content to 6 wt.%, the thermal conductivity reached a peak of 5.17 W/(m·K), which is a substantial 95.83% increase compared to the GO50 sample. This improvement is attributed to the fibrous structure of the AlN whiskers, which act as thermal conduction bridges, connecting the isolated Al_2_O_3_ particles and enhancing heat transfer throughout the material. Nevertheless, when the AlN whisker content surpassed 6 wt.%, the thermal conductivity of the sintered sheets declined to 4.16 W/(m·K). This decrease may be due to the rapid reduction in density, as depicted in Figure 8e, where the ceramic matrix becomes filled with air, reducing the matrix density and impeding the formation of a conductive network. The presence of air voids disrupts the continuous thermal pathways, thus lowering the overall thermal conductivity of the composite.

### 3.4. Dielectric Properties

**Figure 11:**
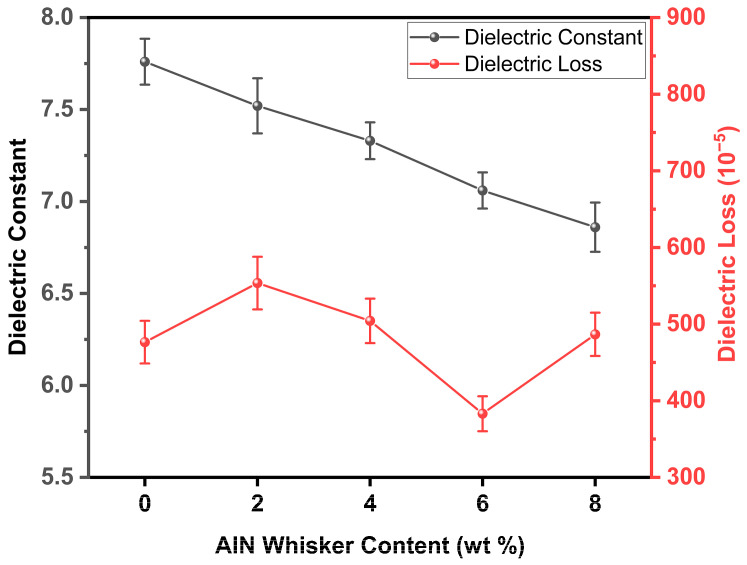
The dielectric properties of composite materials.

Figure 11 illustrates how the dielectric constant (ε_r_) and dielectric loss (tanδ) of the GOWx composites change with the incorporation of AlN whiskers at a frequency of 10 GHz (air conditioning system that maintains a constant temperature of 25 °C and a relative humidity of 50%). The low dielectric constant of aluminum nitride allows AlN whiskers to effectively reduce the dielectric performance of the composites. The introduction of AlN whiskers resulted in a gradual increase in the porosity of the composites post-sintering, as depicted in Figure 8. This increase in porosity, in turn, contributed to a reduction in the dielectric constant. Therefore, the dielectric constant exhibited a monotonic decrease with the incorporation of AlN whiskers. Furthermore, the presence of certain nucleated crystalline phases, like anorthite, can also reduce the dielectric constant [34,35,36,41]. Consequently, the dielectric loss was minimized (tanδ = 3.83 × 10^−3^) when the AlN whisker content was 6 wt.%. There are several factors that may potentially influence the dielectric loss of the composites across varying AlN whisker contents. When a small amount of whiskers is added (e.g., GOW2), numerous heterogeneous interfaces are formed in the composite, which tend to increase the dielectric loss [42]. As the whisker content increases from 2 to 6 wt.%, the formation of an anorthite/AlN whisker network enables some electromagnetic waves to propagate through a three-dimensional network with reduced conduction loss, thereby decreasing the dielectric loss. However, when the whisker content further increases to 8 wt.%, the dielectric loss abruptly increases, possibly due to the presence of large stresses at the pores, which generate defects in the lattice of the material. The larger the number of such defects in the composites, the higher the dielectric loss [43].

Table 4 summarizes the differences in performance between GO50 and GOW6 specimens. Although there was a slight decrease in mechanical properties, they still maintain at a considerable level. The experiment revealed that the most optimal dielectric properties and thermal properties (permittivity of 7.06, dielectric loss of 383.04 × 10^−5^, and thermal conductivity of 5.17 W/(m·K)) were achieved in the GOW6 composite sintered at 800 °C. Notably, the thermal conductivity increased by 95.83% compared to the undoped specimen, while the dielectric constant and dielectric loss decreased by 9.02% and 19.61%, respectively.

## 4. Conclusions

In this work, CMZBS/Al_2_O_3_/AlN composites containing varying amounts of AlN whiskers were fabricated through a process involving sintering between 700 °C and 1000 °C. The work examined their densification behavior, dielectric properties, thermal expansion, flexural strength, and thermal conductivity, with particular attention given to the crystallization behaviors during the sintering process. The addition of AlN whiskers encourages the formation of anorthite phases, which contribute to enhancing the dielectric properties and thermal conductivity of the material. Moreover, an appropriate amount of AlN whiskers can help form a three-dimensional thermal conduction network, effectively improving thermal conductivity. These results highlight the potential of the composite for applications in LTCC technology. However, further work is needed to optimize the distribution of AlN whiskers to enhance the overall performance of the composite materials.

## Figures and Tables

**Table 1:** Composition of CMZBS glass raw materials.

Component	Content (wt.%)	Production Company	Purity
CaCO_3_	33.20	Macklin Reagent Co., Ltd., Shanghai, China	99.99%
MgO	7.38	Macklin Reagent Co., Ltd., Shanghai, China	99.99%
ZnO	7.38	Macklin Reagent Co., Ltd., Shanghai, China	99%
H_3_BO_3_	26.21	Aladdin Biochemical Technology Co., Ltd., Shanghai, China	99.9%
SiO_2_	25.83	Aladdin Biochemical Technology Co., Ltd., Shanghai, China	99.99%

**Table 2:** Characteristics of ceramic raw materials.

Component	Density (g/cm^3^)	CTE (10^−6^/K)	Thermal Conductivity (W/m·K)	Specifications (μm)
Al_2_O_3_ Powder	4.0	7.5	30	D_50_ = 0.5
AlN Whiskers	3.2	4.5	319	Diameter = 0.3, Length = 3~15

**Table 3:** The composition of composite materials used in this study.

Sample Code	Glass (wt.%)	Al_2_O_3_ (wt.%)	AlN Whiskers (wt.%)
GO50	50	50	0
GOW2	49	49	2
GOW4	48	48	4
GOW6	47	47	6
GOW8	46	46	8

**Table 4:** Comparison of physical properties of GO50 and GOW6 specimens.

Properties	GO50	GOW6
Density (g/cm^3^)	3.00	2.89
Flexural Strength (MPa)	242	180
Thermal Conductivity (W/(m·K))	2.64	5.17
CTE (10^−6^/K)	6.80	6.40
Dielectric Constant	7.76	7.06
Dielectric Loss (10^−5^)	476	383

## Data Availability

The original contributions presented in this study are included in the article. Further inquiries can be directed to the corresponding author.
